# Crown-Ether Functionalized Graphene Oxide Membrane for Lithium Recovery from Water

**DOI:** 10.3390/membranes12020233

**Published:** 2022-02-18

**Authors:** Luisa Baudino, Alessandro Pedico, Stefano Bianco, Monica Periolatto, Candido Fabrizio Pirri, Andrea Lamberti

**Affiliations:** 1 Politecnico di Torino, Dipartimento di Scienza Applicata e Tecnologia (DISAT), Corso Duca Degli Abruzzi, 24, 10129 Torino, Italy; alessandro.pedico@polito.it (A.P.); stefano.bianco@polito.it (S.B.); monica.periolatto@polito.it (M.P.); fabrizio.pirri@polito.it (C.F.P.); andrea.lamberti@polito.it (A.L.); 2 Istituto Italiano di Tecnologia, Center for Sustainable Future Technologies, Via Livorno 60, 10144 Torino, Italy

**Keywords:** lithium extraction, raw material recovery, graphene oxide, GO membrane, crown ether

## Abstract

The massive worldwide transition of the transport sector to electric vehicles has dramatically increased the demand for lithium. Lithium recovery by means of ion sieves or supramolecular chemistry has been extensively studied in recent years as a viable alternative approach to the most common extraction processes. Graphene oxide (GO) has also already been proven to be an excellent candidate for water treatment and other membrane related applications. Herein, a nanocomposite 12-crown-4-ether functionalized GO membrane for lithium recovery by means of pressure filtration is proposed. GO flakes were via carbodiimide esterification, then a polymeric binder was added to improve the mechanical properties. The membrane was then obtained and tested on a polymeric support in a dead-end pressure setup under nitrogen gas to speed up the lithium recovery. Morphological and physico-chemical characterizations were carried out using pristine GO and functionalized GO membranes for comparison with the nanocomposite. The lithium selectivity was proven by both the conductance and ICP mass measurements on different sets of feed and stripping solutions filtrated (LiCl/HCl and other chloride salts/HCl). The membrane proposed showed promising properties in low concentrated solutions (7 mg_Li_/L) with an average lithium uptake of 5 mg_Li_/g in under half an hour of filtration time.

## 1. Introduction

The interest in lithium during the past years has been steadily increasing. Its application in secondary batteries may be last in chronological terms, but already accounts for two thirds of the world market of this critical element. The global lithium-ion battery market size is projected to grow from USD 41.1 billion in 2021 to USD 116.6 billion by 2030 [1]. Other applications include ceramics and glasses, lubricating greases, and polymer production [2]. Unfortunately, mineral resources do not grow easily unlike the market demand, and they are rapidly decreasing in both quantity and grade [3,4,5]. A solution to this supply problem has been partly achieved with the soda-lime evaporitic process of natural salt lakes [6]. However, these concentrated basins are few in number nor easily accessible, and the extraction is both time and water consuming. As an alternative to the evaporitic technique, many processes have been proposed to harvest lithium from less concentrated and more widespread water resources such as wastewater and seawater [7,8,9,10,11,12]. In this case, the main issues in harvesting lithium reside in the fact that the compositions of these basins greatly differ, with lithium concentrations ranging from 0.17 ppm in the case of seawater to several hundred ppm in wastewater and brines. Furthermore, while lithium is scarcely present, other monovalent and divalent cations such as sodium and magnesium are abundantly present, making the selectivity of the recovery methods of critical importance [11,13]. The adoption of the recovery process is therefore heavily influenced by the composition and the overall characteristics of the feed solutions. Among these processes, one can differentiate two main categories of lithium recovery techniques: passive processes based on physico-chemical bonding of the cation to a sorbent, and electrochemical processes in which an electric flow is applied. The first category is generally used for more diluted streams and comprises the use of ion sieves [14], supramolecular systems, membrane techniques such as ultra and nanofiltration [15,16], and liquid–liquid extraction [17]. On the other hand, electrochemical processes [13,18] including ion pumping, capacitive deionization, and electrodialysis systems [19] are generally used in pretreated brines of high concentration in more automated setups.

This work itself focuses on an ion imprinted membrane (i.e., a supramolecular system). The selectivity of crown ethers toward cations as a function of their ring size has been extensively proven in both theoretical and experimental works in the past fifty years. Smaller rings such as the four and five membered ones have been proven to selectively bind with smaller cations such as lithium and sodium and reject the bigger ones through steric hindrance [20,21,22,23,24,25,26,27,28,29]. Works of dual crown ether functionalized to successfully recover two different cations at once have also be reported [30]. The family of 12-crown 4-ethers is known to be extremely selective toward lithium [31,32,33,34,35,36,37,38] due to the perfect fit of its cavity with the ionic radius of the lithium ion and is therefore used in this approach. Recently, their possible application in lithium recovery has been investigated by grafting them on polymers or graphene oxide to realize either ion-imprinted polymers or membranes [30,35,36,39,40,41,42,43]. Several polymers have been tested for these kinds of applications, the most common being poly(vinylidene fluoride) (PVDF) [39,40,42] and polysulfone [44] or natural polymers [37].

Graphene oxide (GO) membranes have widely attracted the interest of the research community because of their ease of preparation and functionalization, together with their renowned separation properties [31,45,46,47,48,49,50,51,52]. While GO has been extensively used in membranes for oil–water separation and other water treatments, it is generally not used as the main material in lithium recovery purposes, but only as additive [40,42]. However, its sub-nanometric hydrophilic channels, rich in oxygen groups that can be tuned and functionalized to tailor its filtration properties and its antifouling properties make them appealing for the separation or targeting of ions in water streams [53,54]. Furthermore, the tunability of the channels between graphene oxide flakes can be enhanced when preparing the membrane under vacuum or pressure assisted filtration, as can be carried out in dead-end filtration setups. This kind of preparation has been shown to provide highly stacked membranes, the optimal morphology for filtration and separation purposes [48,54,55]. Furthermore, a dead-end filtration apparatus can provide faster filtrations and is an easier technology to scale-up in industrial pilot scales. The use of such a setup could therefore be the answer to some of the main issues of adsorption processes such as the length of the experiments and their difficult automation.

In this work, the lithium selectivity of a highly stacked GO membrane functionalized with a 12-crown-4-ether molecule is presented. The mechanical properties of the membrane have been improved by using a polymeric binder and the membranes were thoroughly characterized. Although graphene oxide has been previously functionalized with crown ether molecules, it has rarely been used in a stacked morphology for these kinds of applications. Furthermore, the application of an additional pressure can reduce the time of the processes and therefore their costs. A pressure-driven setup has therefore been used to assess the suitability of this kind of membrane to harvest lithium from diluted aqueous resources that are abundant and generally require further treatments before lithium recovery can be undertaken. Conductivity and inductively coupled plasma (ICP) mass measurements were performed and its selectivity with respect to sodium, the most abundant monovalent cation in seawater, was also demonstrated, thus making the membrane an interesting candidate for lithium extraction in fairly diluted streams.

## 2. Materials and Methods

### 2.1. Membrane Preparation

Graphene oxide (GO) flakes were functionalized by carbodiimide esterification by adapting a procedure previously published [42]. Fifty mg of commercial GO (single layer GO, 300–800 nm of lateral dimension, Cheap Tubes Inc., Cambridgeport, VT, USA) were dispersed in dimethyl sulfoxide (DMSO, >99.5%, Sigma Aldrich, St. Louis, MO, USA) and sonicated for one hour at 40 kHz. No centrifugations were performed to further select the size of the GO flakes. N,N’-dicyclohexylcarbodiimide (DCC, >99%, Sigma Aldrich, St. Louis, MO, USA) and 4-(dimethylamino)pyridine (DMAP, >99%, Sigma Aldrich, St. Louis, MO, USA) were then added in a 10:20:1 weight ratio of GO:DCC:DMAP in DMSO as catalysts for the esterification. After sonication of 30 min, a second solution of 200 mg of 2-hydroxymethyl-12-crown-4 (2M12C4E, >95%, Sigma Aldrich, St. Louis, MO, USA) in 10 mL of DMSO (previously sonicated for 1 h) was added and the resulting solution was finally sonicated for 2 h at 40 kHz. The flakes functionalized following this procedure were called f-GO.

A polymeric binder was then added with a concentration of 40 wt% and the solution was sonicated for 30 min more. Different binders were tested: poly(vinylidene fluoride) (PVDF, M_w_ 534000, Sigma Aldrich, St. Louis, MO, USA), poly(vinyl alcohol) (PVA, M_w_ 31,000–50,000, 98–99%, Sigma Aldrich), and pristine GO. The f-GO mixed with the binder was then extracted from the solvent through centrifugation with acetone and transferred to deionized water and sonicated for 30 min at 40 kHz. The membranes were obtained through vacuum assisted filtration on a polycarbonate support membrane (Whatman^®^ Nuclepore™ Track-Etched Membranes, diameter 47 mm, pore size 0.1 μm). No further treatment was performed to polymerize or chemically graft the PVA, which therefore only acted as a physical spacer between the flakes to stabilize them.

### 2.2. Membrane Characterization

Morphological, structural, and physico-chemical characterizations of the membranes were performed on both the nanocomposite membrane and membranes of pristine GO and f-GO (without binders). Field emission scanning electron microscopy (FESEM Supra 40, Zeiss) was used to investigate the morphology of the stacked flakes and their interaction with the binder. X-ray diffraction (XRD) measurements (Panalytical X’Pert MRD Pro X-ray diffractometer with a Cu Kα source) were used to obtain the interplanar distance of the GO and f-GO flakes. The measurements were performed on dry samples prior to the filtration tests with a scan speed of 200 s/step and a step size of 0.026° in Bragg–Brentano configuration. 

The surface chemical properties of the membranes and the success of the functionalization were investigated by Z potential measurements on aqueous solutions with a GO concentration of 0.05 mg/mL by Zetasizer Nano ZS90 (Malvern Panalytical, Malvern, UK). The Raman spectrum was recorded on a Renishaw InVia micro-Raman with an excitation laser of 785 nm of wavelength. The acquisition was performed at 5% of the nominal power and 20× microscope objective. Fourier transform infrared (FTIR) measurements were performed on a Nicolet 5700 FTIR, by Thermo Fisher Scientific, Waltham, MA, USA. The analyses were performed directly on the membranes in an attenuated total reflection (ATR) configuration using a scan speed of 6.33 cm/s and a step size of 0.4 cm^−1^. Thermogravimetric analyses (TGA) were performed on approximately 8 mg of each membrane in 100 μL alumina pans on a Mettler Toledo TGA1 instrument. A heating rate of 10 °C min^−1^ was applied under a nitrogen atmosphere (rate 50 mL min^−1^).

### 2.3. Filtration Properties

The membrane was first characterized by obtaining its permeability curve. Specific amounts of deionized water were filtered at increasing pressure under nitrogen gas in a dead-end pressure filtration setup (Sterlitech HP4750), as reported elsewhere [48]. The selectivity of the membrane was then evaluated in the same dead-end pressure filtration setup of 4.7 cm of diameter under 5 bar of nitrogen gas. All tests were conducted on the nanocomposite membranes and on pristine GO membranes and f-GO membranes without binders as a means of comparison. These were conducted by successively filtrating 15 mL of lithium chloride (LiCl, >99%, Sigma Aldrich, St. Louis, MO, USA), sodium chloride (NaCl, >99.5%, Sigma Aldrich, St. Louis, MO, USA), potassium chloride (KCl, >99%, Carlo Erba), magnesium chloride (MgCl_2_ > 98%, Sigma Aldrich, St. Louis, MO, USA), calcium chloride (CaCl_2_ > 99%, Sigma Aldrich, St. Louis, MO, USA), and 1 mM and 15 mL of hydrochloric acid (HCl, 37%, Sigma Aldrich, St. Louis, MO, USA) 100 mM, respectively, to capture and release cations. Deionized water was flushed through the chamber between each filtration in order to avoid contamination. The solutions were analyzed after five filtration cycles and each type of membrane was tested for at least three filtrations cycles. Each solution was analyzed three times and an average and standard deviation was performed on the results. Conductivity measurements (Edge Conductimeter, Hanna Instruments) were performed on the solutions as they were and ICP mass measurements were performed on samples of the same solutions diluted 1:1000 and digested with a diluted nitric acid solution. The analyses were performed with a Thermo Scientific iCAP Q ICP-MS (Thermo Fisher, Waltham, MA, USA) and data were collected and processed by the related Thermo Fisher ICP Software.

## 3. Results

### 3.1. Membrane Fabrication

**Figure 1:**
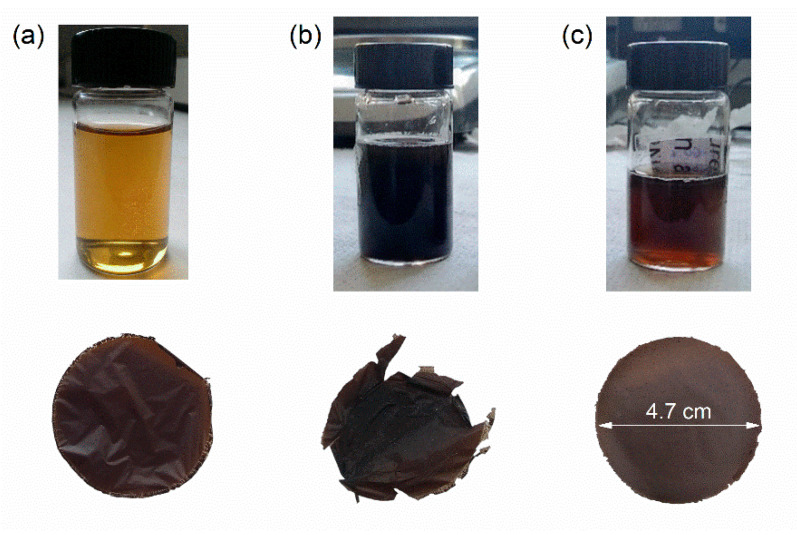
Images of the formulations and f-GO membranes with the binders (**a**) GO, (**b**) PVDF, and (**c**) PVA.

Good permeability and stability of the membranes are some of the key parameters to achieve high performances in separation and harvesting processes. An initial concentration of functionalized GO flakes was chosen equal to 0.2 mg/mL, in order to achieve a high dispersion in deionized water. However, these membranes resulted in being fairly brittle. Following a previously published work [48], the initial concentration of GO flakes was then increased to 1 mg/mL and several binders were also considered to improve the mechanical properties of the resulting membranes. Pristine GO, PVDF, and PVA were chosen as binders in light of their already extended use in water treatment applications [56,57,58]. The resulting membranes can be seen in Figure 1. Whereas pure GO and PVDF needed higher loads to achieve mechanical stability, PVA proved to be the most suited binder for this kind of application. The resulting membrane was stable and easily peelable from its support with 40 wt% of binder, whereas the other membranes crumpled and resulted in being fairly brittle. Furthermore, due to its hydrophilicity, PVA should facilitate the passage of ions and therefore improve the filtration properties [43,56]. The stability of the membrane was also assessed by leaving it in an aqueous solution for more than three months, after which the membrane showed no sign of degradation.

**Figure 2:**
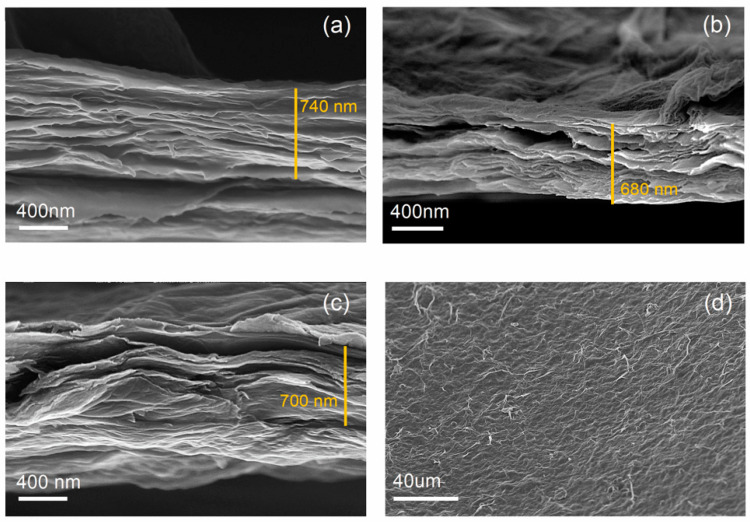
FESEM images of (**a**) pure GO membrane, (**b**) f-GO membrane, (**c**) nanocomposite membrane, and (**d**) top view of the nanocomposite (f-GO+PVA) membrane.

The nanocomposite membrane comprising f-GO flakes and PVA obtained under pressure in the dead-end apparatus was further characterized before testing its properties during filtration. A morphological characterization through electron microscopy was performed in order to assess the uniformity of the stacked flakes and whether there was a good interaction between the binder and the f-GO flakes. Figure 2 shows a comparison between the morphologies of pristine GO membrane, a f-GO membrane, and the nanocomposite membrane. All three showed similar thicknesses and good stacking of the GO flakes. The nanocomposite membrane also showed good homogeneity, indicating that the binder was successfully incorporated into the structure.

### 3.2. Membrane Characterization

**Figure 3:**
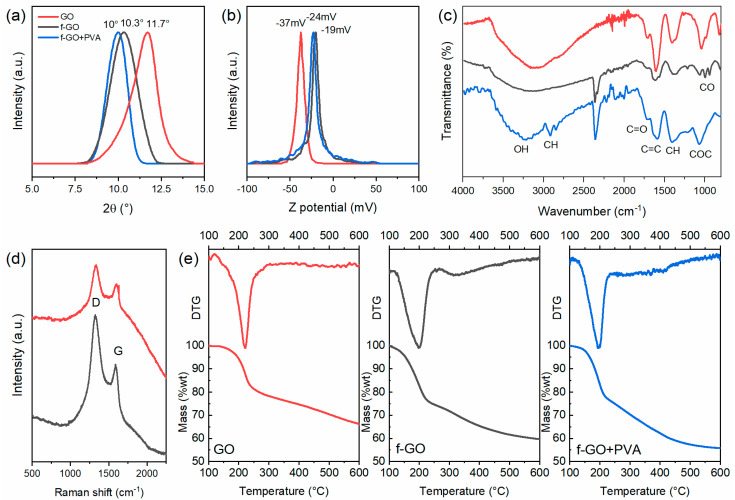
Characterizations of the three different membranes where the pristine GO is the red line, the f-GO is the black line, and the nanocomposite is the blue line. (**a**) XRD patterns of the three membranes prior to the filtrations. (**b**) Z-potential measurements of the aqueous solutions. (**c**) FTIR spectra, (**d**) Raman spectra, and (**e**) TGA and DTG of the three membranes.

Several characterizations were performed on the membranes to investigate their surface properties. The interplanar distance of the three membranes was obtained through XRD measurements on dry samples prior to the filtrations, focusing on the (001) diffraction peak of GO around 10°. Figure 3a shows that the peak shifted to lower values when going from pure GO to the functionalized and nanocomposite. By using Bragg’s law, the average interplanar distance increased from 7.6 Å of pristine GO to 8.2 Å and 8.9 Å when it was functionalized and PVA was present, respectively. This is in good accordance with the literature reporting that the crown ether molecule should be between the flakes and act as a spacer between the channels [53].

Z-potential measurements (Figure 3b) and FTIR analyses (Figure 3c) were performed to investigate the superficial charge and the functional groups of the membranes. The first showed that although the three membranes were all negatively charged, hence attracting cations, the functionalized ones had smaller charges, being partially reduced. The peaks were located at −37 mV, −19 mV, and −24 mV for pristine GO, f-GO, and f-GO+PVA, respectively, which are in good accordance with the literature [48,59]. FTIR analyses (Figure 3c) evidenced the presence of the crown ether molecule by the presence of the peaks between 1100 cm^−1^ and 950 cm^−1^ related to the C–O and O–C–O stretching vibrations in the aromatic ring, as described elsewhere [38,42,43,60]. The GO characteristic broad absorbance band of O–H stretching vibrations was also visible between 3500 cm^−1^ and 3000 cm^−1^, and the C=O and C=C vibration frequency at 1720 cm^−1^ and 1620 cm^−1^ were also appreciable, as was the band at 2940 cm^−1^ of the C–H alkyl groups of PVA [61]. The Raman spectra (Figure 3d) instead showed the typical peaks of GO: the D peak was observable at 1324 cm^−1^ and G peak at 1585 cm^−1^. The Raman active modes of the crown ether molecules, which would be between 1250 and 1300 cm^−1^ and between 1450 cm^−1^ and 1500 cm^−1^ were covered by the GO ones, which are more intense [60].

The partial reduction in GO flakes as a result of the functionalization and the presence of the crown ether molecule was further evidenced by the thermal analyses (TGA, Figure 3e), here reported with their differential (DTG). It can be seen that the more volatile molecules caused a decrease in the onset of the thermal degradation from 220 °C of the pristine GO to around 200 °C for the two functionalized ones.

### 3.3. Filtration Properties and Selectivity

**Figure 4:**
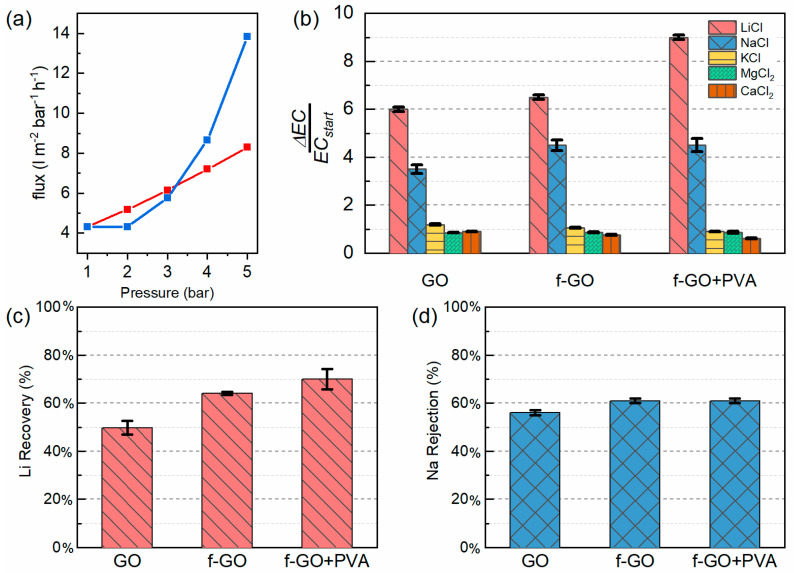
(**a**) Permeability curve of the GO (in red) and f-GO+PVA (in blue) membrane, (**b**) conductivity measurements, (**c**) lithium recovery rate, and (**d**) sodium rejection rate from ICP mass analyses of the three membranes. The conductivity measurements were performed in the salt solutions whereas the ICP mass measurements were carried out on the stripping solutions.

The membranes were then further tested in the pressure assisted dead-end filtration setup with deionized water. Initially, the water flux as a function of the pressure was investigated. As can be seen in Figure 4a, the pristine GO membrane presented a linear flux, whereas the other increased in a non-linear way. The operating pressure was chosen equal to 5 bar following this trend for all membranes.

The filtration properties of the membranes were then evaluated for chloride salts with the five cations most present in seawater and commercially extracted (i.e., sodium, potassium, magnesium, calcium, and lithium) [62]. A concentration of 1 mM for each salt was chosen to simulate the conditions of fairly diluted streams. Although the lithium concentration in seawater and freshwater is two orders of magnitude lower than this chosen concentration (i.e., 0.17 ppm ca), 1 mM seemed to be a good compromise between freshwater and the first stages of lithium enrichment, which usually needs to be undertaken prior to lithium extraction. Indeed, diluted lithium solutions generally need to be pretreated before lithium extraction to reach higher concentrations and facilitate the extraction processes [7,8,63]. On the other hand, the concentration of the eluent is the result of a compromise between the need for a sufficiently low pH for the regeneration of the crown ether molecules and the minimization of the use of corrosive solutions. The choice, therefore, resulted in a concentration of 0.1 M.

The lithium adsorption mechanism here implemented consisted of (i) lithium immobilization into the crown ether ring during the filtration of the salt and (ii) the release of lithium under acidic conditions when the crown ether molecules were regenerated during the HCl filtration. Although crown ether molecules have been generally used in batch adsorption conditions, several works have reported their efficiency under flow conditions [53,64]. This could lead to a possible scaling up of continuous lithium enrichment and extraction processes, thus reducing the costs and duration at the same time. Each couple of filtrated solutions of salt/HCl were characterized by conductivity measurements. The conductivity of each solution was measured at 25 °C under magnetic stirring (100 rpm) after each filtration. Three values were taken for each solution and an average was performed. The increase in conductivity between the last cycle and the initial feed for each solution is reported in Figure 4b. Although the measurements were taken for both the feed solution (salts 1 mM) and the stripping solution (HCl 100 mM), only the values for the feed solution were taken into consideration as the most representative ones. This choice was motivated by the significantly higher conductivity of the acid solution with respect to the salts (even in equimolar conditions). Therefore, when the exchange H^+^/Li^+^ takes place in the crown ether molecule, the conductivity of the salt solution should increase, whereas that of the acidic solution should decrease. Since this change is more evident in the salt solution, which has a lower starting conductivity, the delta was computed on it. The results were calculated as follows:

(1) $$\frac{\Delta \mathrm{EC}}{{\mathrm{EC}}_{\mathrm{start}}}=\frac{\mathrm{Final}\;\mathrm{solution}\;\mathrm{conductivity}-\mathrm{Initial}\;\mathrm{solution}\;\mathrm{conductivity}}{\mathrm{Initial}\;\mathrm{solution}\;\mathrm{conductivity}}$$

As can be seen in Figure 4b, the change in conductivity of all the membranes tested was significant only in the case of the sodium and lithium ions, which were the ones most similar in terms of size and could therefore compete in the complexation, together with the crown ether ring. ICP mass measurements were performed on the stripping solutions of these two sets of solutions in order to assess the reliability of the conductivity measurements by means of the target cation concentration (see Figure 4c,d). The recovery rate of lithium was calculated as:

(2) $$\mathrm{Lithium}\;\mathrm{recovery}\;\mathrm{rate}=\frac{\mathrm{Final}\;\mathrm{stripping}\;\mathrm{solution}\;\mathrm{concentration}}{\mathrm{Initial}\;\mathrm{feed}\;\mathrm{solution}\;\mathrm{concentration}}\times 100$$

whereas the rejection rate of sodium was considered and computed as

(3) $$\mathrm{Sodium}\;\mathrm{rejection}\;\mathrm{rate}=\frac{\mathrm{Initial}\;\mathrm{feed}\;\mathrm{concentration}-\mathrm{Final}\;\mathrm{stripping}\;\mathrm{concentration}}{\mathrm{Initial}\;\mathrm{feed}\;\mathrm{solution}\;\mathrm{concentration}}\times 100$$

By looking at the graphs, it is possible to see that the membranes are indeed selective toward lithium, with a significant improvement in the performances of the functionalized membranes. In the case of the pristine GO membranes, the uptake of lithium and sodium resulted from the electrostatic bonding between the cations and the negative surface of the membrane. Indeed, in this case, the two cations were adsorbed in equal measure. Instead, in the case of functionalized GO, the selectivity of the membrane increased. Furthermore, the presence of the binder in the nanocomposite membrane not only ensures a better stability of the membrane, but also provides similar results to the case of f-GO, with the Li^+^/Na^+^ separation coefficient ranging between 1.64 and 1.79. Each membrane was tested for several filtration cycles and there was no evidence of any degradation in the successive filtration cycles, thus proving the stability of the setup.

Seventy percent of the total amount of lithium could be recovered by acid stripping after five filtration cycles, resulting in an overall recovery of 24.5 mg_Li_/g_membrane_ and an average of 4.9 mg_Li_/g_membrane_ for each filtration cycle. This uptake was lower than those previously reported, namely 24.25 mg_Li_/g_membrane_ in the case of the PVDF membrane with functionalized GO flakes from [40] or the 27.55 mg_Li_/g_membrane_ of the polyether sulfone from [41]. However, the previously reported results were obtained with higher initial lithium concentration, namely from 50 mg_Li_/L [41,42] to 1000 mg_Li_/L [43], and during longer times. Indeed, this work was able to significantly shorten the duration of the process from several days (the conventional time for batch adsorption) to just a few hours while working with a 7 mg_Li_/L solution. If one were to calculate an efficiency of lithium recovery, the solution herein proposed would reach 70% of the total amount of lithium recovered after five cycles, whereas the previous studies could recover around 30% of the initial amount. Furthermore, this study used lower concentrations of acidic solutions to decrease the impact of the process and reported smaller adsorption times since one filtration only took 30 min compared with the batch adsorption processes, which required from several hours to some days.

## 4. Conclusions

This work described a method to produce f-GO membranes for lithium recovery. A crown ether selective toward lithium was grafted onto GO flakes through carbodiimide esterification. A polymeric binder was successfully mixed with the f-GO to improve the mechanical properties, after which the membranes were prepared by vacuum filtration to obtain a homogeneously stacked morphology of the GO flakes. Filtration tests confirmed that the functionalization was responsible for an increase in the selectivity of the membranes toward lithium, improving the rejection of sodium and other monovalent and divalent cations. Moreover, adding the polymeric binder resulted in a positive synergy with the f-GO, increasing the uptake of lithium, while slightly reducing that of calcium and did not affect the behavior toward NaCl or other chloride salts.

In the configuration herein proposed, an impressive lithium recovery rate of 70% was reached in just a few hours from a diluted stream of 7 mg_Li_/L after five consecutive filtration cycles, with an average lithium uptake of 4.9 mg_Li_/g_membrane_ for each cycle. This work sheds further light on the possibility of using graphene oxide membranes for lithium recovery from diluted water sources, pushing forward the research in a field that is nowadays of worldwide interest.

## Data Availability

Data is contained within the article.
